# Translation and adaptation to Brazilian Portuguese of the Lymphedema Rating Scale in Head and Neck Cancer

**DOI:** 10.1590/S1679-45082017AO3995

**Published:** 2017

**Authors:** Débora dos Santos Queija, Lica Arakawa-Sugueno, Bruna Mello Chamma, Marco Aurélio Vamondes Kulcsar, Rogério Aparecido Dedivitis

**Affiliations:** 1 Curso de Pós-Graduação em Fisiopatologia Experimental, Faculdade de Medicina, Universidade de São Paulo, São Paulo, SP, Brazil; 2 Faculdade de Medicina, Universidade de São Paulo, São Paulo, SP, Brazil; 3 Universidade de Mogi das Cruzes, Mogi das Cruzes, SP, Brazil; 4 Instituto do Câncer do Estado de São Paulo, São Paulo, SP, Brazil

**Keywords:** Lymphedema, Head and neck neoplasms, Neck dissection, Radiotherapy, Lymph nodes, Validation studies, Linfedema, Neoplasias de cabeça e pescoço, Esvaziamento cervical, Radioterapia, Linfonodos, Estudos de validação

## Abstract

**Objective:**

Translate to brazilian portuguese, culturally adapt and test the rating and classification scales of cervicofacial lymphedema of the MD Anderson Cancer Center Head and Neck Lymphedema Protocol (MDACC HNL) in patients undergoing treatment for head and neck cancer.

**Methods:**

The process followed international guidelines and translation stages by two head and neck surgeons, and back translation independently by two native Americans. The test of final version was based on the evaluation of 18 patients by one speech pathologist and one physical therapist who applied the scales in Portuguese.

**Results:**

The translation of the three scales was carried out independently and the translators reached a consensus for the final version. Minor modifications were made by translating two terms into the Assessment of the Face. Versions of back-translation were similar to each other. The instrument was successfully applied to patients independently.

**Conclusion:**

The translation and cultural adaptation of the assessment and rating scale of the cervicofacial lymphedema of the MD Anderson Cancer Center Head and Neck Lymphedema Protocol to the Brazilian Portuguese were successful.

## RESUMO

**Objetivo:**

Traduzir, para o português brasileiro, adaptar culturalmente e testar as escalas de avaliação e classificação do linfedema cérvico-facial do MD *Anderson Cancer Center Head and Neck Lymphedema Protocol* (MDACC HNL) em pacientes submetidos ao tratamento para o câncer de cabeça e pescoço.

**Métodos:**

O processo seguiu as diretrizes internacionais e as etapas de tradução por dois cirurgiões de cabeça e pescoço, além de retrotradução de forma independente por dois nativos norte-americanos. O teste da versão final foi realizado a partir da avaliação de 18 pacientes por um fonoaudiólogo e um fisioterapeuta, por meio da aplicação das escalas em português.

**Resultados:**

A tradução das três escalas foi realizada de forma independente, e os tradutores chegaram a um consenso para a versão final. Foram feitas pequenas modificações, ao serem traduzidos dois termos em *Assessment of the Face* . As versões da retrotradução foram semelhantes entre si. O instrumento foi aplicado com sucesso nos pacientes de forma independente.

**Conclusão:**

A tradução e a adaptação das escalas de avaliação e classificação do linfedema cérvico-facial do MD *Anderson Cancer Center Head and Neck Lymphedema protocol* para o português foram bem sucedidas.

## INTRODUCTION

The modalities of head and neck cancer treatment have the objective of control of the disease and patient survival. Both the surgical approach (with or without neck dissection) and exclusive or adjuvant radiation therapy or associated with chemotherapy aim to cure and preserve the function of the structures involved in respiration, voice, swallowing, and as much as possible, to maintain the integrity of the patient's face and neck.^(^ ^1^ ^,^ ^2^ ^)^ Although treatment increases survival, there is a great risk of patients developing acute and/or chronic sequelae, with an important impact on quality of life.^(^ ^3^ ^–^ ^5^ ^)^

Some of the complications inherent to the treatment are secondary edema and lymphedema. Surgical manipulation can rupture the lymphatic structures and damage the adjacent soft tissues, leading to an increase of lymphatic fluid in the interstitial spaces. Lymphatic fluid retention activates inflammatory and immunological responses, resulting in fibrosis of soft and subcutaneous tissues, as well as in adipose deposition, which hinders lymphatic function.^(^ ^6^ ^,^ ^7^ ^)^ Postoperative edema can recede in a question of days, while edema caused by the rupture of lymphatic and vascular drainage can remain and take months to disappear. The toxicity of radiation also promotes damage that leads to alterations during and after treatment, compromising the blood vessels and the lymphatic channels of the face and neck, contributing to lymphedema.^(^ ^3^ ^,^ ^8^ ^,^ ^9^ ^)^

Lymphedema can involve external anatomical sites (soft tissues of the face and neck). As a consequence of external lymphedema, swelling can lead to reduced range of movement, pain, and discomfort in the neck.^(^ ^2^ ^,^ ^3^ ^,^ ^5^ ^,^ ^10^ ^)^

Despite lymphedema being recognized as a significant complication of head and neck cancer treatment, it still is underdiagnosed and undertreated. The literature suggests a prevalence that varies from 54 to 75%.^(^ ^10^ ^,^ ^11^ ^)^ In the last few years, some authors have been concerned about describing and characterizing lymphedema in this population, but the studies are not sufficiently enlightening.^(^ ^12^ ^)^

The medical literature indicates that the understanding of the pathological mechanisms points out that lymphedema and fibrosis occur continuously. Some patients present with swelling, while others may develop fibrosis without necessarily having a previous history of edema. However, many patients evolve with both, and fibrosis represents the final stage.^(^ ^13^ ^,^ ^14^ ^)^ Both events are generally associated with functional impairment and therefore, it is essential there be a tool that allows identification, evaluation, and measurement of its severity.^(^ ^15^ ^,^ ^16^ ^)^

Some protocols have been developed and tested throughout this decade with the purpose of characterizing secondary lymphedema, fibrosis, and its respective impact on the functional condition and on the quality of life of these patients, and as a treatment aid, but there still is no consensus.^(^ ^6^ ^,^ ^8^ ^,^ ^10^ ^,^ ^12^ ^,^ ^13^ ^,^ ^15^ ^–^ ^26^ ^)^

Only the MD Anderson Cancer Center (MDACC) group published and has used scales that allow measurements of cervicofacial lymphedema in head and neck cancer.^(^ ^12^ ^,^ ^24^ ^,^ ^26^ ^)^ The tool named MD Anderson Cancer Center Head and Neck Lymphedema (MDACC HNL *)* has been applied in other studies and compared to other instruments.^(^ ^14^ ^,^ ^21^ ^)^ The MDACC HNL Protocol includes the interview with the patient, the visual and tactile evaluation of the face, neck and shoulders region, and the functional assessment of swallowing and oral communication. The examination also combines photography, measurement with a measuring tape, and staging of the edema to characterize the general appearance and severity of the lymphedema.^(^ ^12^ ^)^

## OBJECTIVE

Translate to brazilian portuguese, culturally adapt and test the rating and classification scales of cervicofacial lymphedema of the MD Anderson Cancer Center Head and Neck Lymphedema Protocol (MDACC HNL) in patients undergoing treatment for head and neck cancer.

## METHODS

This research represents the initial phase of the clinical study project, approved by the Research Ethics Committee of the Medical School – *Universidade de São Paulo,* where it was carried out under number 137/14, from January to December 2016. To develop the project with the three scales, the author gave her permission to translate it. The Assessment of the Face, Neck Circumference, and the MDACC Head and Neck Lymphedema Rating Scale, are all parts of the MD Anderson Head and Neck Lymphedema Protocol.

Since these are strictly anatomy-related measurement scales, the translation was done independently by two head and neck surgeons with experience in head and neck edema and lymphedema, and proficiency in the English language, based on *Nomina Anatomica* .^(^ ^27^ ^)^ The process was based on international guidelines.^(^ ^28^ ^,^ ^29^ ^)^

There was, therefore, a consensus between the translators of a new version for Brazilian Portuguese and the posterior back-translation, performed independently by two native English language speakers. Next, the back-translation was compared with the original, analyzing aspects related to conceptual equivalence, semantics, and content. Later, a translation was prepared by the committee formed by the translators and back-translators.

The authors recommend that the lymphedema assessment be applied by a professional certified in lymphedema.^(^ ^12^ ^,^ ^25^ ^)^ The application of the final version was done independently by two healthcare professionals (one speech therapist and one physical therapist) certified by the Leduc method, by means of perimetry adapted for face and neck, and a visual and tactile evaluation, in order to stage the lymphedema. The patient was assessed individually by each one of the professionals who then compared the evaluations. Since they are similar measurements, the evaluators reached a consensus.

All patients were photographed, as recommended by the authors, using as background a checkered frame, with a Canon EOS T4i camera, and an 18-55mm objective lens.^(^ ^12^ ^)^ The instruments were applied to 18 patients of the Head and Neck Service of the organization where the study was carried out.

### Evaluation of the facial edema or lymphedema

**Figure 1:**
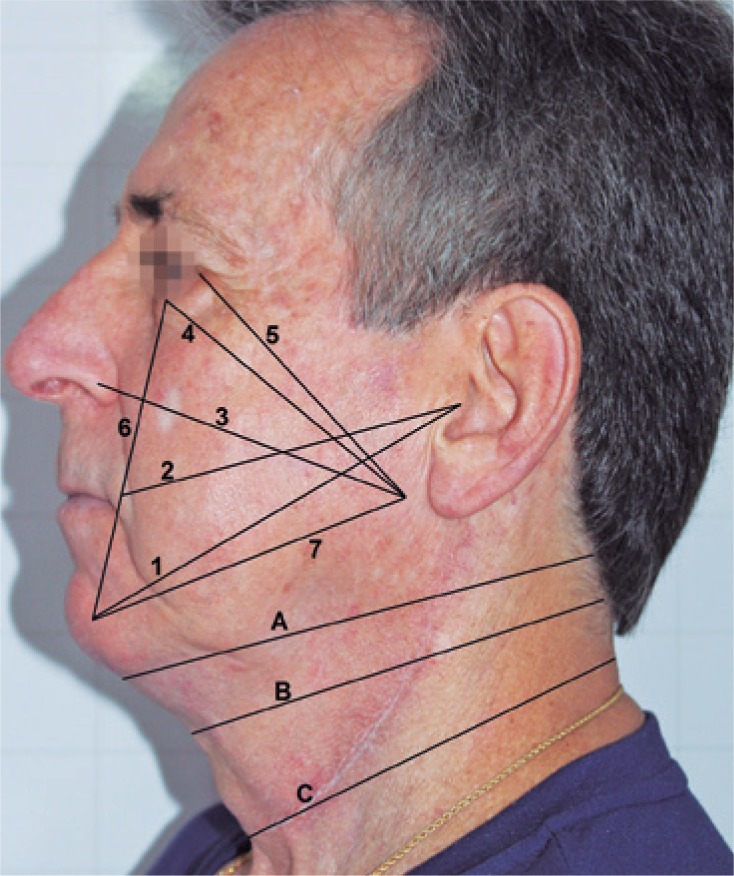
Measurements assessing cervicofacial edema or lymphedema

For the visual evaluation, the patient's face was marked with an antiallergenic pen to bilaterally measure the facial proportions ( Figure 1 ), using adapted perimetry (anthropometric measuring tape), as per the criteria adopted by Smith et al., which has two facial circumference measurements (diagonal and submentonian), point to point, and seven measurements that characterize the facial composite.^(^ ^12^ ^)^

### Lymphedema staging

The MD Anderson Cancer Center Head and Neck Lymphedema Rating Scale was created to characterize the presentation and severity of lymphedema in head and neck surgery, and it is based on the traditional Földi Scale for staging of lymphedema in the extremities. On the adapted MDACC scale, level 1 was divided into 1a and 1b to describe the edema as pitting and non-pitting. In this case, the evaluation is made by digital pressure on the skin of the edema area, which indicates the presence of interstitial fluid in the region. Pressure was exerted softly on the evaluated area for a period of 10 seconds. The edema is considered as pitting when there is identification of the tissue depression remaining after the digital pressure. The depth of the tissue depression and the time it remains reflect the severity of the edema. Pitting edema is softer, while non-pitting edema is more rigid and does not yield to pressure.^(^ ^12^ ^,^ ^14^ ^)^

## RESULTS

The translation of the three scales of the MDACC HNL Protocol was performed by two head and neck surgeons ( Chart 1 ). Both translations were analyzed together by the two translators who reached a consensus for the final version.

**Chart 1:** Original instrument, translations, consensus, and back-translation

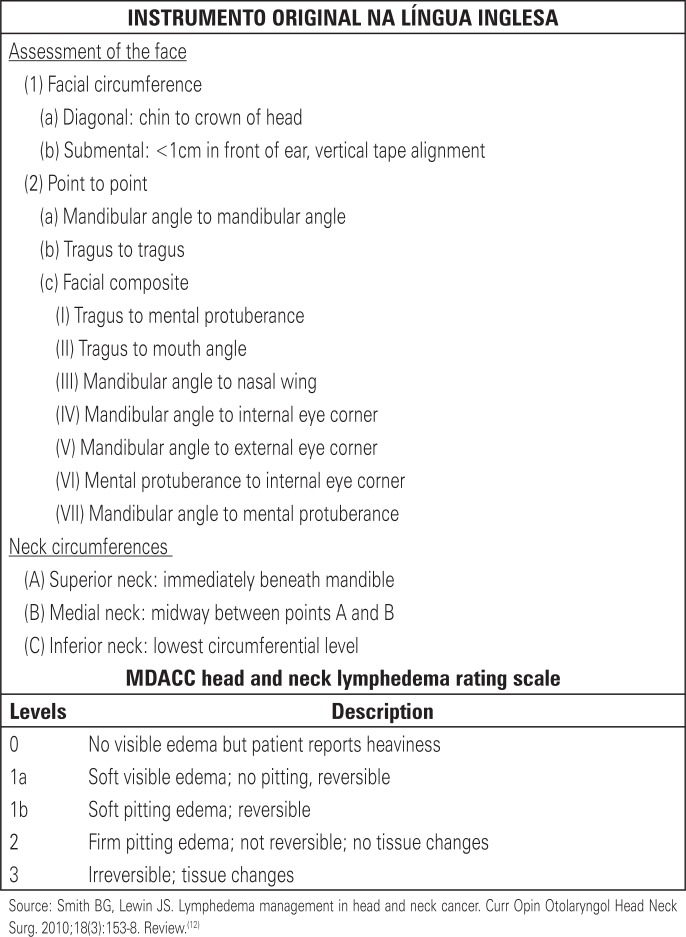
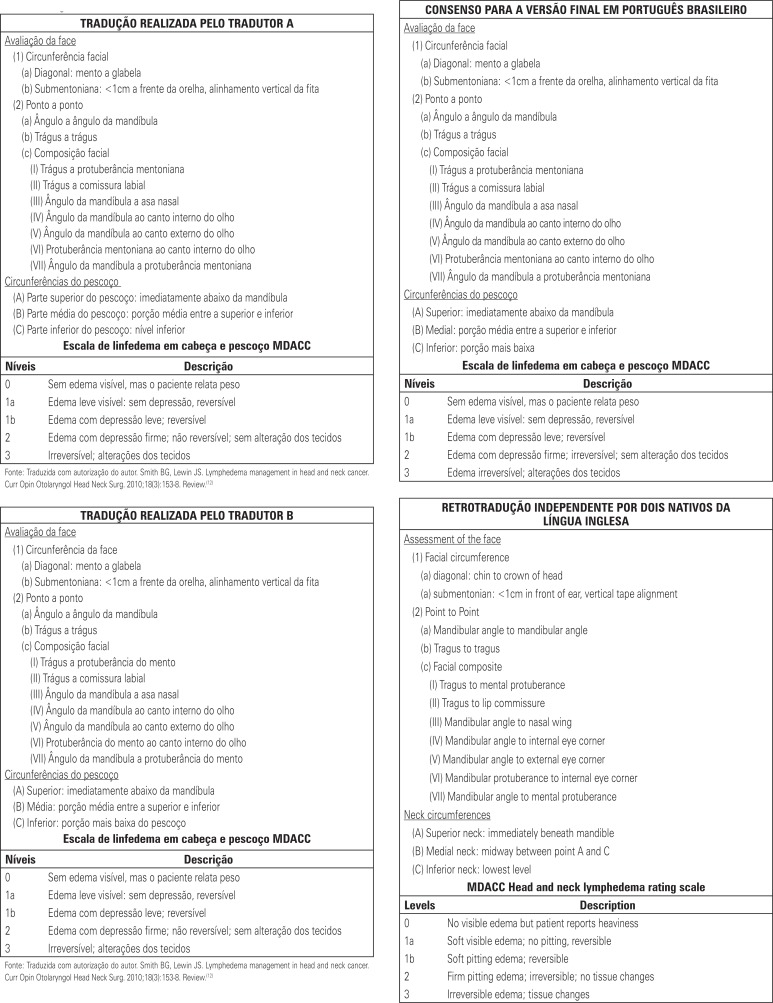

The translators made small modifications when translating two terms in the Assessment of the Face. The first was “crown of head,” which when translated into Portuguese, was called “glabella.” The second was “mouth angle,” which was called “labial commissure.” The adaptation did not compromise the content, from the semantic point of view. Additionally, the said scales should be applied by healthcare professionals with experience in anatomical terminology of the head and neck.

Based on this last version, the back-translation was done independently by two bilingual translators. The versions were similar between themselves, with no impairment of the original version.

The instrument was applied by the healthcare professionals on 18 patients at the organization where this research was carried out. Table 1 shows the characterization of cases in which the translated scales were applied. The study subjects had lesions in various sites, and the treatment used varied from surgery to radiation therapy and chemotherapy, and 15 underwent neck dissection.

**Table 1:** Demographic, clinical, and treatment characteristics

Variable		n
Age (years)		
	Minimum-maximum	36-82
	Median	60
	Mean±standard deviation	61.22±11.39
Sex		
	Female	6
	Male	12
Tumor location		
	Mouth	7
	Oropharynx	5
	Larynx	1
	Infraglottic cavity	1
	Thyroid	1
	Face	2
	Occult primary tumor	1
Staging		
	Tx	1
	T1b	1
	T2	10
	T3	2
	T4	2
	N0	10
	N1	2
	N2	1
	N2a	2
	N2b	1
Treatment		
	Surgery	8
	Surgery + radiation therapy	4
	Surgery + radiation therapy and chemotherapy	5
	Radiation therapy and chemotherapy	1
Neck dissection		
	No	3
	Yes	14
Type of neck dissection		
	Supraomohyoid	9
	Radical	3
	Modified radical	1
	Jugular	1
	Selective	1
Radiation therapy		
	Minimum-maximum	3,150-7,000
	Median	1,575
	Mean±standard deviation	3,186±3,292.57
Interval since end of treatment (months)		
	Minimum-maximum	3-40
	Median	6.5
	Mean±standard deviation	11.94±12.12
Alcoholism		
	No	18
	Yes	-
Smoking		
	No	16
	Yes	2
Tracheostomy		
	No	17
	Yes	1
Nasogastric tube		
	No	17
	Yes	1

In the group evaluated, facial lymphedema was identified in ten patients (55.5%), four of them (40%) type 1a, six (60%) type 1b. Table 2 indicates data related to facial measurements.

**Table 2:** Assessment of the face

Patient	1a	1b	2a	2b	Measurements (mm)													
					CI		CII		CIII		CIV		CV		CVI		CVII	
					R	L	R	L	R	L	R	L	R	L	R	L	R	L
1	17	29	21.5	34	11	11.5	11	10.5	11.5	11	13.5	12.5	7	7.5	12	12.5	10	9.5
2	13	26	24	27.5	13.5	14	9.5	10	10	11	13	13.5	9	9	10.5	10.5	12	12.5
3	13.5	31	28	32	16	16	12	12	13	14	15.5	16	12	12	11.5	11.5	14	14
4	13	28	22	29	16	15.5	11.5	11.5	11.5	12	12	12.5	9.5	9	10.5	11	11	11
5	14	30	26.5	31	17	17.5	13	12.5	12.5	11	14.5	13.5	9.5	9.5	12	12	14	12.5
6	15.5	28	20	30	16	15.5	12	11	11	10	12	12	9	9	12	12	10	10
7	15	27	29	29	15.5	16	10	11	10	10	13	13	10	9	12	12	19	10
8	17	29	17	23	16	17	12.5	12	12	12	14.5	14.5	11	11	14	14	11.5	10
9	14.5	27.5	19.5	32	15	15	12	12	11	9	13	11	9	9	11	11	10	9.5
10	12	23	18	30	16.5	17	11	12	12	11.5	12	13	10	10	11.5	11.5	12	13.5
11	14	30	20	30	16.5	17	12.5	12	12	12	14	14.5	11	10	12	11.5	11.5	13
12	15	27	21.5	28	14	14	11	11	10	10	12	12	10	10	11	11	9	9
13	14	27	19.5	28	15	17	10	11	10	10	12	12	8.5	10	12	12	8	10
14	15	27	18	29	15.5	14.5	11	10	10	11	12.5	12.5	9.5	9	11.5	11	9.5	11
15	15	32	25	32	17	17	11.3	11.3	11	11	15.5	14	12	12	15	14	10	11.7
16	13	26	17.5	28.5	15	15.5	11	11	9	9	12	12	10	10	12	12	9	9
17	14	31	20	30	17	17	12	12	10	10	13	13	10	10	12	12	10	10
18	19	30	18	29	16	16	11	11	10	10	13	13	11	11	12	12	9	9

mm: millimeters; 1a diagonal: mentum to glabella; 1b submentonian: <1cm to the front of the ear, vertical tape alignment; 2a: angle to mandibular angle; 2b: tragus to tragus; CI: tragus to mentonian protuberance; CII: tragus to labial commissure; CIII: mandibular angle to nasal wing; CIV: mandibular angle to the internal corner of the eye; CV: mandibular angle to the external corner of the eye; CVI: mentonian protuberance to the internal corner of the eye; CVII: mandibular angle to the mentonian protuberance; R: right; L: left.

Table 3 shows the neck measurements. Fourteen (77.7%) patients presented with lymphedema of the neck, six of them (42.8%) type 1b reversible, and eight (57%) of the irreversible type. In this latter group with irreversible lymphedema, five (62.5%) were type 2, and three (37.5%) were type 3.

**Table 3:** Measurements of the neck

Patient	Measurements (mm)		
	Superior	Medial	Inferior
1	43.5	41	40
2	36	39	36.5
3	40	40.5	40
4	36	35	36
5	41	39.5	41
6	42.5	40	41
7	36.5	35	36.5
8	42	40	39
9	43	40	43
10	38	37.5	37.5
11	39	40	40.5
12	39	38	38
13	37.5	35	35.5
14	37	34	35
15	46	46.5	44
16	36	34	36
17	40.5	42	44
18	38.2	36	36.5

## DISCUSSION

The basic concern in seeking rating scales that allow assessment of lymphedema and fibrosis after treatment for head and neck cancer have shown that there are specificities and differences in each one. Some focus on the aspects related to edema *per se* , while others explore the characteristics of fibrosis.^(^ ^7^ ^)^ The Földi et al., Scale^(^ ^13^ ^)^ seems to capture the changes in soft tissues, from the softest and reversible edema, to the firmest one, which is not reversible.^(^ ^14^ ^)^ Even so, it is difficult to measure these alterations, a fact that justifies the scarce number of publications on the topic, and a consensus that the existing rating scales are inconsistent in capturing the characteristics of the external lymphedema after head and neck cancer.

In its initial phase, often the lymphedema can be difficult to detect. However, with the use of measuring techniques, it is possible to identify lymphatic congestion and increased pressure in the subcutaneous tissue. If the intervention is performed early, primarily in cases in which the damage is severe, the results can be satisfactory.^(^ ^6^ ^,^ ^12^ ^,^ ^24^ ^)^

These scales have already been used by some authors and had as criterion of choice previous experience demonstrated in studies with the instrument.^(^ ^18^ ^–^ ^20^ ^,^ ^25^ ^)^ The staging and evaluation scales of cervicofacial lymphedema were standardized by the MDACC, and are a part of the evaluation protocol of head and neck cancer lymphedema of the organization; they are also associated with the functional evaluations of swallowing and with the patient's follow-up.^(^ ^12^ ^)^ The use of the anthropometric measuring tape for the adapted perimetry is mentioned because of the difficulty in establishing the reference points that allow constant and reproducible results.^(^ ^18^ ^,^ ^19^ ^,^ ^23^ ^)^ By the number of patients studied in the samples and by inconsistency of the points used in measuring, there still is no evidence in the method, but it is the form available for this purpose. It is recommended that a photographic registry be made of the front and profile views, with the patient sitting and the camera positioned on a tripod, always in the same location. We also suggest the attachment of a checkered frame on the wall behind the patient and the demarcation, with an appropriate pen, so that the record of the image is made always in the same position. This trick facilitates follow-up and the accompaniment and progress of the edema and lymphedema throughout treatment.

Recently, a protocol was drawn up with a few assessment measurements of lymphedema of head and neck cancer (ALOHA). The instrument consists of measurements of the perimetry of the superior and inferior circumferences of the neck, ear to ear, and of the extension of the lip to the inferior circumference of the neck (border of the lower lip to the inferior circumference of the neck). In addition to these, an instrument was used to evaluate the content of tissue fluid in the face and of the neck (Moisture MeterD) by means of a probe. The study indicated a weak reliability of the measurements extracted from the extension of the lip to the inferior circumference of the neck, while the comparison between the staging scale and the constant dielectric constant of the tissue achieved good reliability. The perimetry measurements of the tissue were useful to assess the changes in the tissue throughout time, and the Moisture MeterD was effective to diagnose lymphedema.^(^ ^19^ ^)^

The description, presentation of the external lymphedema, and results of complete decongestive therapy were verified by means of the measurements obtained with the rating scales and the MD Anderson Cancer Center Head and Neck Lymphedema Protocol cervicofacial lymphedema in 1,202 patients treated for head and neck cancer. The study identified a predominance of external 1b lymphedema in 62% of patients, followed by 28% of type 1a. The rating scale helped identify the changes obtained after treatment. The authors commented that the subdivision of level 1 into 1a and 1b improved the ability to capture the visible non-pitting edema, which is common in head and neck cancer cases, and that is not commonly found in patients with lymphedema in the extremities.^(^ ^24^ ^)^

The results of this project pointed out the presence of external facial lymphedema in 55.5%, and of the neck in 77% of the assessments, indicating similarity with the findings in literature.^(^ ^6^ ^,^ ^11^ ^)^

Despite the fact that they do not characterize diagnostic tools, when applying the MD Anderson Cancer Center Head and Neck Lymphedema Protocol evaluation and classification rating scales of cervicofacial lymphedema, it was possible to observe that the perimetry measurements offer an aid for the capture of the volume of edema and external lymphedema.^(^ ^12^ ^)^

For the application of the scales to be fruitful, it is advisable that the instrument by applied by speech therapists, physical therapists, and physicians who already have experience, as well as certification in the identification and management of edema and lymphedema in head and neck cancer.^(^ ^12^ ^,^ ^25^ ^)^

The study highlighted some limitations related to grading of the fibrosis. So far, when the involvement is greater, there are no validated instruments that can characterize the several more subtle degrees of tissue changes. Nevertheless, even so, the instrument used allowed conditions favorable to perceiving the differentiation between pitting and non-pitting lymphedema, such as in the cases found in head and neck cancer.

With the rating scales of external lymphedema, it is possible to incorporate systematic evaluation into the routine of patient progress. The information obtained by means of the MD Anderson Cancer Center Head and Neck Lymphedema Protocol rating scales for evaluation and classification of cervicofacial lymphedema can enable early detection and identification of lymphedema, besides providing support to referral to treatment, while at the same time, serving as an instrument for assessing the patient's clinical progress.

## CONCLUSION

The translation of the MD Anderson Cancer Center Head and Neck Lymphedema Protocol rating scales for evaluation and classification into Portuguese was consistent with the original texts.
